# Percutaneous Total Endoscopic Resection of Partial Articular Processes for Treatment of Lateral Crypt Stenosis and Lumbar Spinal Stenosis: Technical Report and Efficacy Analysis

**DOI:** 10.1155/2018/9130182

**Published:** 2018-10-18

**Authors:** Fujun Wu, Weijun Kong, Wenbo Liao, Jun Ao, Sheng Ye, Qian Du, Ansu Wang

**Affiliations:** Department of Spinal Surgery, The First Affiliated Hospital of Zunyi Medical College, Zunyi 563000, China

## Abstract

**Objective:**

To observe the clinical curative effect of posterior total endoscopic precision decompression for the treatment of single-segment lateral crypt lumbar spinal stenosis (LSS).

**Method:**

A total of 27 patients with single-segment LSS satisfying the inclusion criteria were recruited from July 2013 to September 2015. There were 18 cases of unilateral stenosis of the L_4-5_ segments and 9 cases of unilateral stenosis of the L_5_-S_1_ segment. All patients were treated via the posterior approach with the precise lateral crypt decompression technique. Precise decompression was performed on the narrow areas causing clinical symptoms. Clinical efficacy was assessed at 3 days, 3 months, 6 months, and 2 years after surgery. Low-back pain and sciatic nerve pain assessed by visual analog scale (VAS) score and the functional Oswestry Disability Index (ODI) were used to evaluate lumbar function, and modified MacNab score criteria were used to investigate long-term efficacy.

**Result:**

All patients completed the operation successfully, and the follow-up time was 2 years. The VAS score of lumbago was lower after than before surgery (preoperative: 6.96±0.90; postoperative: 2.04±1.02, P<0.05). The VAS score of sciatica was also lower after than before surgery (preoperative: 7.19±0.88, postoperative: 1.93±0.92, P<0.05), and the ODI was improved at the last follow-up (29.62±4.26) % compared with before surgery (80.07±3.98) %. The MacNab efficacy evaluation showed improvement at the end of the follow-up period: 20 cases were excellent, 6 cases were good, and 1 case was satisfactory, with a good/excellent rate of 96%. No surgical site infections, iatrogenic nerve root injuries, epidural hematomas, or other complications occurred.

**Conclusion:**

Total endoscopic decompression of posterior facet arthrodesis for the treatment of single-segment lateral crypt LSS has the advantages of safety, reduced recurrence and trauma, and a satisfactory curative effect. This trial is registered with ChiCTR1800015628.

## 1. Foreword

**Figure 1:**
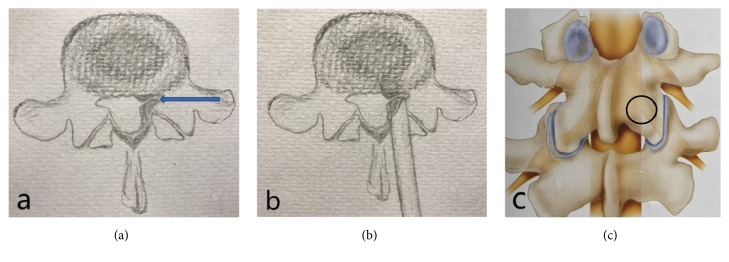
(a) Left lateral crypt stenosis. (b), (c) Operational area of arthroscopic and total endoscopic precision decompression techniques.

Lumbar spinal stenosis (LSS) is one of the most common degenerative diseases encountered in spinal surgery. According to the anatomical classification, there are four types of LSS: central, lateral, intervertebral, and intervertebral foramen [1]. The lateral recess type is due to its special anatomical position; located in the upper articular process, the anterior and posterior walls are limited, and the anterior wall is formed by the fibrous ring of the intervertebral disc, while the posterior wall is formed by the small joint [2]. The most common pathological basis of lateral crypt stenosis is hypertrophic osteoarthritis, followed by intervertebral disc fibrosis and endplate osteophyte growth [3, 4] (Figure 1(a)). The lateral recess is located at the entrance of the nerve root into the nerve root canal. Lateral recess stenosis leads to compression of the nerve roots, which is associated with the presence of lumbago and/or sciatica as clinical symptoms. Early-stage patients usually use NSAIDs, physiotherapy, nerve blocks, or epidural steroid injections, along with changes in their lifestyle, multidisciplinary rehabilitation, and other conservative treatments [5]. Surgical treatment is needed for patients in whom conservative treatments are not effective [6]. The purpose of surgical treatment is to decompress the lateral recess and relieve the nerve roots. A variety of surgical procedures have been used to treat lateral recess stenosis, ranging from standard open laminectomy to minimally invasive decompression [7–15]. Traditional treatment methods mainly focus on complete decompression, such as by opening the laminar window, decompressing the vertebral disc, and removing the intervertebral disc. Sometimes, the ideal therapeutic effect can be achieved by intervertebral fusion or internal fixation [16, 17]. However, total laminectomy decompression of the spinal cord destroys the stability of the spine, and lumbar instability, spondylolisthesis, and chronic low-back pain may occur after surgery [18]. In recent years, minimally invasive techniques to access the lumbar vertebrae, intervertebral disc mirrors, endoscopic techniques, and minimally invasive intervertebral foramen bone graft fusion, among others, have been developed and widely applied in the treatment of LSS and have achieved good clinical effects [19, 20]. Among them, percutaneous endoscopic decompression (PED) is an effective decompression under the endoscope for reduced volume of spinal canal due to degeneration, including the treatment of herniated discs, hypertrophied and ligamentum flavum, and facet joints, narrow lateral recesses, or nerve root canals. PED has gradually become a common treatment method for lumbar spinal canal stenosis [21]. PED technology is mainly based on the narrow area, and the approach can be either via the intervertebral entry or intervertebral orifice. The intervertebral foramen approach can be used to decompress lateral recess stenosis at the intervertebral pore level. There is no effective approach for the decompression of lateral recess stenosis at the pedicle level. At the same time, due to blocking by the high iliac bone in some patients, 20% of patients are not suitable for endoscopic decompression using the intervertebral foramen approach. Because of the special anatomical position of the lateral crypt, it is necessary to remove part of the articular joint to achieve complete decompression endoscopically operation. Additionally, to achieve a vertebral plate gap for endoscopy, a high-speed grinding drill or a laminar rongeur can be used to remove part of the inside edge of the laminar or articular process; the endoscopic operation range is small, and the lateral crypt presents certain difficulties to relieving the stress of this condition. We treated patients with L_4-5_, L_5_-S_1_ single-segment lateral LSS by applying posterior facet arthrodesis total endoscopic crypt decompression (Figures 1(b) and 1(c)), and we obtained a good clinical curative effect.

## 2. Materials and Methods

### 2.1. General Information

The inclusion criteria were as follows: aged 56 to 73 years presenting with low-back pain, lower-limb radiative pain, muscle weakness, and/or paresthesia; computed tomography (CT) and/or magnetic resonance imaging (MRI) results indicating that the affected segment was unilateral L_4-5_or L_5_-S_1_; conservative treatments received for 3 to 6 months.

The exclusion criteria were as follows: cauda equina syndrome leading to complete urinary dysfunction; central spinal canal stenosis; lumbar hyperextension flexion X-rays suggesting lumbar spondylolisthesis or instability in the surgical segment; spinal deformities, spinal fractures, ankylosing spondylitis, spinal tuberculosis, spinal infection, or spinal tumors; symptoms of cervical spondylosis; coagulation dysfunction; and abnormal mental behavior.

**Figure 2:**
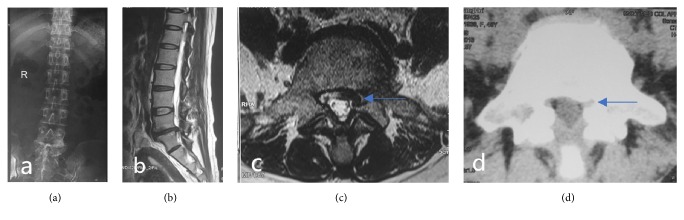
(a) Preoperative lumbar anteroposterior X-ray. (b), (c) Preoperative sagittal MRI T2-weighted images showing L_5_-S_1_ intervertebral disc degeneration and the lack of normal adipose tissue surrounding the left nerve root on the horizontal axis. Stenosis of the lateral crypt (arrows). (d) CT of the transverse axis of the lumbar intervertebral disc before surgery showing hyperplastic cohesion of the left facet joint and narrowing of the superior articular process (arrows).

A total of 27 patients were enrolled according to the inclusion and exclusion criteria. There were 19 males and 8 females aged 56 to 73 years, with an average age of 61.1 years. There were 18 cases involving the L_4-5_ segments and 9 cases involving the single side of L_5_-S_1_. The 27 patients showed low-back pain and sciatica. Twenty-one patients had leg sensory disorders. Lower-extremity muscle weakness was observed in 5 patients, and 1 patient showed decreased tendon reflexes (Figure 2).

### 2.2. Surgical Methods

**Figure 3:**
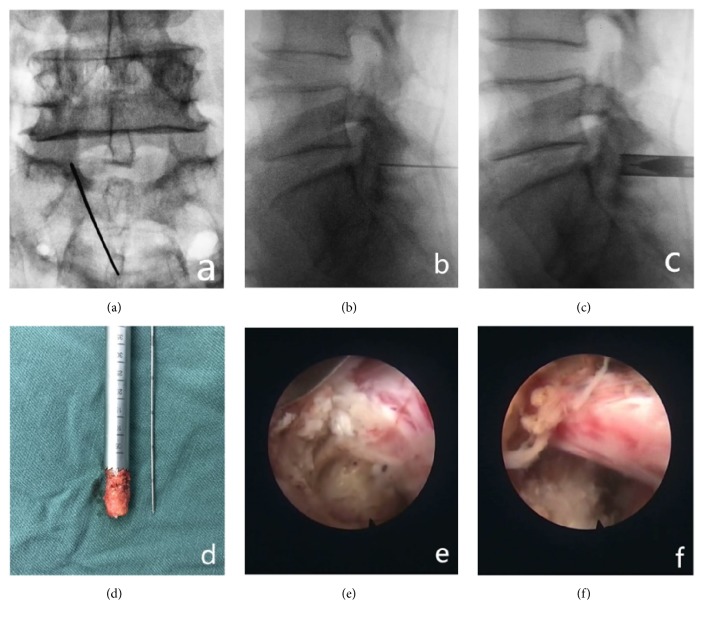
(a), (b) Accurate positioning of lateral crypt stenosis with the following articular process in the center. (c) Part of the lower articular process is removed with a circular saw to establish a working channel. (d) Part of the lower articular process bone removed with a circular saw. (e) Herniated disc tissue is exposed outside the shoulder of the nerve root and removed. At the same time, the posterior edge of the vertebral body is removed, and the nerve root is decompressed. (f) The nerve root is dorsally, ventrally, and laterally decompressed thoroughly, allowing it to relax.

Continuous epidural anesthesia was applied to patients in the prone position. Intraoperative X-ray fluoroscopy was used to locate the lesion segment. The center of the articular process under the segment of interest or the most obvious site of stenosis was targeted by the puncture needle. X-ray fluoroscopy was used to confirm placement of the puncture needle toward the lower articular process. An approximately 6-mm-long skin incision was made with the guide needle. The guide wire was then inserted into the expansion tube and the work sleeve to the joint process. Imaging examination was used to determine the range of articular resection. During the operation, trephine was used to remove the upper 1/4 of the upper vertebral articular process and the upper vertebral articular process. If necessary, part of the lamina was removed to expand the laminar space. The dilation tube was placed in the endoscopic operating system, and thickened or calcified yellow ligaments were removed, relieving the ventral and dorsal compression of the nerve root in the subarticular process. At the same time, the nerve sheath and dural sac are pulled apart using a working sleeve for complete removal of prominent or bulging disc tissue, followed by ventral nerve root decompression. Then, relaxation of the nerve roots is confirmed, hemostasis is achieved by radiofrequency coagulation, and checks are performed for thermocracking and ruptured fiber ring formation. Complete lateral crypt decompression is then verified, the catheter is removed, and the incision is sutured (Figure 3).

### 2.3. Postoperative Management and Rehabilitation

On the 2nd day after the operation, the waist circumference was worn to start the exercise. Do not advocate strenuous activities, 1 to 2 weeks for bed rest. Resume daily activities and normal work after 3 to 4 weeks. Avoid heavy physical labor and move, screw, lift heavy objects, etc. during work. Wear waist circumference when going out or getting up in 3 months after surgery; at the same time, the back muscle function exercise was started to avoid the disuse of the waist muscles.

### 2.4. Clinical and Imaging Indicators

The patients were assessed for low-back pain and leg pain at 3 days, 3 months, 6 months and 2 years after surgery. The Oswestry Disability Index (ODI) was used to evaluate daily behavior, and the modified MacNab score was used to evaluate the clinical effect.

**Figure 4:**
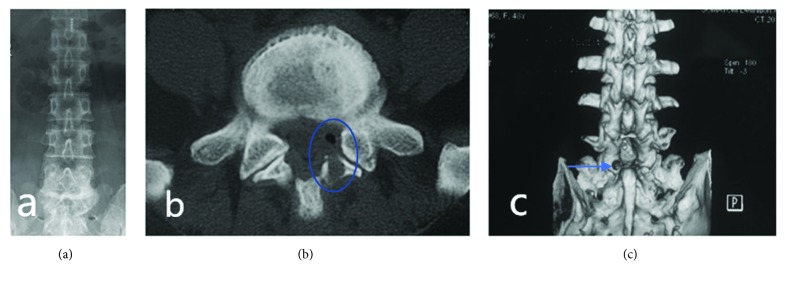
(a) Postoperative lumbar anteroposterior. (b) Posterior lumbar CT showing full left septal decompression. (c) Lumbar vertebra CT + 3D reconstruction showing the articular process sawing window decompression zone (arrows).

Preoperatively, postoperatively and at 2 years after surgery, the patients were examined in terms of the orthopedics of the lumbar spine and overextended flexion. Two years after surgery, lumbar CT scans were reviewed to evaluate decompression of the lateral recess (Figure 4).

### 2.5. Statistical Methods

SPSS 18.0 statistical software was used for statistical processing, and the measurement data are presented as $\overset{-}{x}\pm s$. The visual analog scale (VAS) and ODI scores were assessed repeatedly for the analysis of variance. To measure the degree of activity of the surgical segment before and after the operation, repeated measurements were performed for variance analysis. For all tests, the level of significance was in the form of alpha=0.05.

## 3. Results

The operation time ranged from 90 ~ 130 min, and no patient procedures were converted to open surgery. Patients with low-back pain and sciatica showed significant improvement. The VAS score for lumbago and lower-extremity pain was significantly decreased after surgery (P<0.01). There was no significant increase in lumbar pain in patients at the postoperative follow-up, and the postoperative ODI score was significantly improved (P<0.01). There were significant differences between the preoperative and all the postoperative scores. The efficacy evaluation by MacNab score showed improvement at the end of the follow-up period, with excellent in 20 cases, good in 6 cases, and fair in 1 case; the good/excellent rate was 96% (Table 1).

The patients in this group experienced no nerve injuries, dural tears, cerebrospinal fluid leakages, epidural hematomas, intervertebral space infections or other intraoperative and postoperative complications. Follow-up at two years showed no asymptomatic recurrence, lumbar instability, or other such symptoms.

## 4. Discussion

### 4.1. Overview of LSS and Comparison between Traditional Treatment and Total Endoscopic Techniques

Anatomically, the lumbar spinal canal is divided into two regions, the central canal and the nerve root canal. Stenosis may occur in one or both of these areas at the same time. The nerve root canal is divided into three areas: the lateral crypts, intervertebral foramen, and lateral regions of the foramen. The lateral crypts reported in this article are located in the entrance zone of the nerve root into the root canal. The lateral recess is located under the upper facet joint. The anterior wall is formed by the annulus fibrosus of the intervertebral disc. The posterior wall is formed by the facet joint and is restricted by the anterior and posterior walls. The most common pathological basis of lateral crypt stenosis is hypertrophic facet osteoarthritis, followed by bulging of the intervertebral disc annulus, hyperplasia of the vertebral posterior edge, and hypertrophic calcification of the ligamentum flavum, resulting in decreased lateral crypt height and angle. These conditions cause nerve root compression, resulting in low-back pain, lower-limb dysfunction and other clinical manifestations. Traditionally, lateral crypt LSS is mainly treated through open surgery, usually by laminectomy, laminectomy, or extensive facetectomy. Directly under the excision of the vertebral body with bone callus formation, herniated disc and posterior longitudinal ligament materials are removed to obtain annular nerve root decompression. Open surgery provides a clear view, allowing complete decompression and ideal postoperative symptom relief. However, due to structural damage to the posterior column of the spine after open surgery, some patients need lumbar fusion and internal fixation to maintain spinal stability. According to reports in the literature, the greatest problem with lumbar fusion and instrument fixation is that the incidence of adjacent segment involvement after lumbar fusion is significantly higher than that of nonfusion technique [22]. At the same time, because of the age of some patients, the risk of prolonged anesthesia and surgery cannot be ignored [23, 24]. In addition, the side effects of pulling and stripping soft tissue during the operation can lead to prolonged postoperative recovery periods. With the continuous development and improvement of minimally invasive techniques, endoscopic techniques have gradually become one of the main treatment strategies because of their advantages of reduced trauma and faster postoperative recovery. We used the posterior total endoscopic precise decompression technique to treat lateral recess LSS. Good clinical effects were obtained after the operation. The VAS and ODI scores of patients with low-back pain were significantly better after than before surgery, and their clinical symptoms were continuously relieved during one year of follow-up, with negligible low-back pain and ODI scores. Two years after the operation, the results of the MacNab evaluation indicated that the rate of excellent and good results was 96%.

### 4.2. The Application of Total Endoscopic Technique in Degenerative LSS

Current studies in the literature report that a transforaminal approach is used in most patients with degenerative lumbar lateral crypt stenosis. The dorsal lateral nerve root compression was relieved by removing the anterior 1/3 of the superior facet and the anterior part of the lower articular process, while removing the joint capsule and the posterior lateral nerve ligamentum of the nerve root. The endoscope can reach the predural space through the enlarged intervertebral foramen and be used to treat the prominent intervertebral disc tissue and the posterior edge osteophytes of the vertebral body to achieve ventral decompression and achieve a good clinical effect. Research shows that decompression through the transforaminal approach will not affect the structural integrity and biomechanical stability of the lumbar motion segment, with no iatrogenic lumbar segmental instability occurring after surgery. For lumbar lateral recess stenosis, both the transforaminal and translaminar approaches can effectively reduce nerve root pressure. However, some patients are affected by high iliac blockage, transverse process hypertrophy, osteophyte hyperplasia and other factors, especially in the L_5_-S_1_ segment, increasing the difficulty of transforaminal decompression surgery. In addition, because of the inability of the intervertebral foramen approach to treat pedicle lateral stenosis and central spinal stenosis, its range of indications is narrower. The laminar approach can be used to treat central spinal canal stenosis, using the laminar approach may be more reasonable and simpler. During the surgical procedure, we found that precision decompression technology is crucial in the treatment of lateral crypt LSS. During the percutaneous endoscopic operation, because the internal diameter of the working channel of the foramen mirror is only 6 mm, the available intraoperative space is extremely limited. To ensure the same clinical efficacy as open surgery, we must be able to accurately locate the surgical site before surgery in order to accurately target the nerve root compression site. If the position of interest cannot be accurately located, once the working channel is placed, it is difficult to change the surgical area by adjusting the working channel, and the operation becomes very difficult.

### 4.3. Characteristics and Advantages of the Transarticular and Translaminar Approaches

The lateral recess is also called the “Lee entrance zone.” The nerve roots exit the dural sac here and extend outward under the top facet; arthritis of the facet joints is the most common cause of lateral crypt stenosis. For patients with single-stage lateral crypt LSS, which approach should be used: translaminar or transarticular? The lower margin of the vertebral plate and the articular bone were removed using a high-speed grinding drill under fully endoscopic conditions. Operation of the microscopic rongeur is relatively simple and the same as in open surgery, but it greatly reduces the efficiency of the surgery. The use of high-speed intraoperative drilling also presents certain problems. For example, debris generated during high-speed drilling may lead to a blurred view in the microscope, more bleeding occurs when the cancellous bone is polished, the handle is longer, and the stability of the high-speed grinding bit is reduced. Another potential disadvantage is that the operator's judgment of depth requires extensive experience. Due to the special anatomical location and pathological basis of the lateral crypt, this study provides a sufficient theoretical basis for the selection of precise decompression technology for arthrodesis. In the operation, the center of the articular process is positioned with a K-wire, and the ring saw is guided through the K-wire. Under X-ray monitoring, the upper quarter of the inferior facet joint is resected with a circular saw, leaving part of the isthmus and the upper and lower facet arthrosis structures. Isopathic spondylolysis should be avoided. Resection of part of the lower articular process makes the decompression of the lateral recess simpler and more direct and improves the surgical efficiency, which are exactly the differences between the approach in this study and the total endoscopic interlaminar approach to crypt decompression. The surgeon must follow the principle of “minimum trauma” while performing surgical treatment. Intervertebral disc tissue, facet joints, and ligamentum flavum play critical roles in maintaining spinal stability. Thus, surgeons pursue minimally traumatic solutions to clinical problems. Accurate decompression is based on fully endoscopic techniques performed via the interlaminar approach to accurately decompress the area responsible for stenosis. The partial resection of the lower articular process during the operation retained the posterior structure of the spine to a maximum degree, thus maintaining the stability of the lumbar spine, reducing postoperative lumbar instability and facilitating postoperative recovery.

At the beginning of the study, the surgical indications for localized lumbar spinal stenosis were more strictly controlled, a good initial clinical effect was obtained by implementing a full endoscopic precision decompression technique. Our research should consider some potential shortcomings, the number of reported cases was small. To reaffirm the utility of this approach.it needs to be conducted on a larger number of cases. As the research progressed, our team is currently tracking more postoperative cases and comparing them with the results of open spinal decompression surgery.

## 5. Conclusions

From what has been discussed above, for patients with lateral crypt LSS, the total endoscopic precision decompression technique for posterior facet joint arthrodesis can effectively improve the symptoms of low-back and leg pain in patients. With the features of minimal surgical trauma, direct and safe decompression, rapid postoperative recovery, and satisfactory clinical outcomes, this approach can be used as a surgical method for the treatment of lateral crypt LSS.

## Figures and Tables

**Table 1:** VAS and ODI scores of preoperative and postoperative low-back pain and sciatica (n = 27, $\bar{x}\pm s$).

	Preoperative	3 days after surgery	3 months after surgery	6 months after surgery	2 years after surgery
Low-back pain VAS score	6.96±0.90	3.52±0.80*∗*	3.15±0.95*∗*	2.48±0.94*∗*	2.04±1.02*∗*
Lower-limb pain VAS score	7.19±0.88	3.11±0.75*∗*	2.52±0.75*∗*	2.04±0.90*∗*	1.93±0.92*∗*
ODI	(80.07±3.98) %	(58.67±3.68) %#	(38.81±4.94) %#	(34.37±3.72) %#	(29.62±4.26) %#

Note: *∗*P<0.05 compared with preoperative; #P<0.05 compared with preoperative.

## Data Availability

The data used to support the findings of this study are available from the corresponding author upon request.
